# Analysing dengue fever spread in Kenya using the Zero-Inflated Poisson model

**DOI:** 10.4102/jphia.v16i1.781

**Published:** 2025-02-28

**Authors:** Lameck Ondieki Agasa, Faith Thuita, Thomas Achia, Antony Karanja

**Affiliations:** 1 Department of Public and Global Health, Faculty of Health Sciences, University of Nairobi, Nairobi, Kenya; 2 Department of Community Health and Behavioral Sciences, School of Health Sciences, Kisii University, Kisii, Kenya; 3 Institute of Mathematical Sciences, Strathmore University, Nairobi, Kenya; 4 Department of Mathematics, Faculty of Science and Technology, Multimedia University, Nairobi, Kenya

**Keywords:** dengue fever, Zero-Inflated Poisson model, climatic factors, epidemiology, Kenya

## Abstract

**Background:**

Dengue fever (DF), transmitted by *Aedes* mosquitoes, remains a major public health concern in tropical and subtropical regions. Understanding the influence of climatic variables on DF incidence is essential for improving outbreak prediction and control measures.

**Aim:**

This study analysed the impact of climatic factors on DF incidence in Kenya using a Zero-Inflated Poisson (ZIP) model.

**Setting:**

The study focused on DF cases in Kenya from 2019 to 2021.

**Methods:**

A ZIP model was applied to monthly dengue case data and associated climatic variables, such as temperature, rainfall, and humidity. The model addresses over-dispersion and excess zeros in the data, providing a more accurate depiction of DF dynamics.

**Results:**

The ZIP model revealed significant associations between climatic variables and DF incidence. Humidity (β = 0.0578, standard error [s.e.] = 0.0024, *z* = 24.157, *p* < 2e-16) and temperature (β = 0.0558, s.e. = 0.0053, *z* = 10.497, *p* < 0.01) showed a positive relationship with dengue cases, while rainfall (β = –0.0045, s.e. = 0.0003, *z* = –16.523, *p* < 0.01) had a significant negative effect. The over-dispersion test confirmed excess variability in the data (O statistic = 456.3, *p* = 0.004), and the Vuong test supported the use of the ZIP model over a standard Poisson model. Model comparison indicated superior fit for the ZIP model (akaike information criterion [AIC] = 5230.959 vs. 27061.367 for Poisson), effectively accounting for zero-inflation.

**Conclusion:**

The results suggest that higher humidity and temperature favor dengue transmission, while heavy rainfall may disrupt mosquito breeding, reducing cases. These findings provide a basis for targeted public health interventions.

**Contribution:**

This study enhances understanding of DF-climate interactions in Kenya, supporting the application of ZIP modelling for improved disease surveillance and control strategies.

## Introduction

Dengue fever (DF) is a rapidly spreading viral disease transmitted by mosquitoes, and it has become one of the most pervasive infectious diseases worldwide.^1,2^ Dengue fever is caused by the dengue virus (DENV) and it manifests in four serotypes: DENV-1, DENV-2, DENV-3, and DENV-4.^3,4^ It is transmitted primarily by *Aedes aegypti* mosquitoes. Dengue fever presents with symptoms similar to those of influenza, including high fever, severe headache, muscle and joint pain, rash, and a drop in platelet count.^1,3,5^ Dengue fever can escalate to more severe forms, such as Dengue Haemorrhagic Fever (DHF) and Dengue Shock Syndrome (DSS), which are life-threatening and require prompt medical attention.^6^

Globally, DF impacts over 100 million people annually and results in approximately 25 000 deaths, with the majority of fatalities occurring among children.^1,7,8^ The prevalence of DF is widespread across tropical and subtropical regions, affecting more than 100 countries.^6,9^ The DF disease is a major public health concern, particularly in urban and semi-urban areas where the *Aedes* mosquitoes breed in stagnant water.^10,11^

The spread of DF is intricately linked to climatic conditions.^5,12,13^ Temperature,^14^ precipitation,^15^ and humidity^16^ play pivotal roles in the life cycle of *Aedes* mosquitoes and consequently in the transmission of DENV. Warmer temperatures accelerate mosquito development and increase the frequency of blood-feeding behaviours, thereby enhancing the likelihood of dengue transmission.^14,17,18^ Increased precipitation creates more breeding sites for mosquitoes, while high humidity supports their survival and activity. Recent studies have demonstrated that fluctuations in these climatic variables can lead to significant variations in dengue case numbers.^15,19,20^ Higher rainfall and increased temperatures have been associated with outbreaks in various regions.^12,13,14,15,16,17,18,19,20^ Understanding these relationships is crucial for predicting and managing dengue outbreaks, as climate conditions directly influence mosquito populations and viral transmission dynamics.

To investigate the impact of climatic variables on DF, researchers have frequently employed Poisson regression models. These models have been used to analyse the effects of temperature, rainfall, and other meteorological factors on dengue incidence. For example, studies in Sri Lanka, Indonesia, and other dengue-endemic areas have utilised Poisson regression to explore the relationship between climatic factors and dengue outbreaks.^21,22,23^

In Sri Lanka, a Poisson regression model examined meteorological parameters influencing dengue spread in Colombo between 2010 and 2018, revealing significant associations between climatic conditions and dengue incidence.^21^ Similarly, in Bandung, West Java, Indonesia, a Poisson regression model was used to forecast dengue cases based on temperature and cumulative rainfall data.^22^ These models have provided valuable insights into how climatic variables affect dengue transmission, but they often face limitations, particularly when dealing with excess zeros and over-dispersion in the data.

Traditional Poisson regression models assume that the variance of count data is equal to the mean, a condition often violated in epidemiological datasets due to excess zeros, where many locations or time periods report no cases.^24,25^ This mismatch complicates the use of Poisson regression, as its assumptions do not align with observed data patterns, leading to inaccurate estimates and misinterpretations of disease dynamics. The Zero-Inflated Poisson (ZIP) model addresses these limitations by combining a Poisson distribution with an additional component that explicitly models the excess zeros. This dual approach distinguishes between two processes: one generating excess zeros and another producing non-zero counts, providing a more accurate representation of the data and revealing hidden patterns in disease distribution.^26,27,28^ Successfully applied in various fields, including epidemiology, the ZIP model enhances the understanding of data characterised by excess zeros. For DF, it can identify factors influencing both the likelihood of observing zero case reports and the variability in non-zero case counts, leading to more precise estimates of disease incidence and improved predictions of future outbreaks.

**FIGURE 1:**
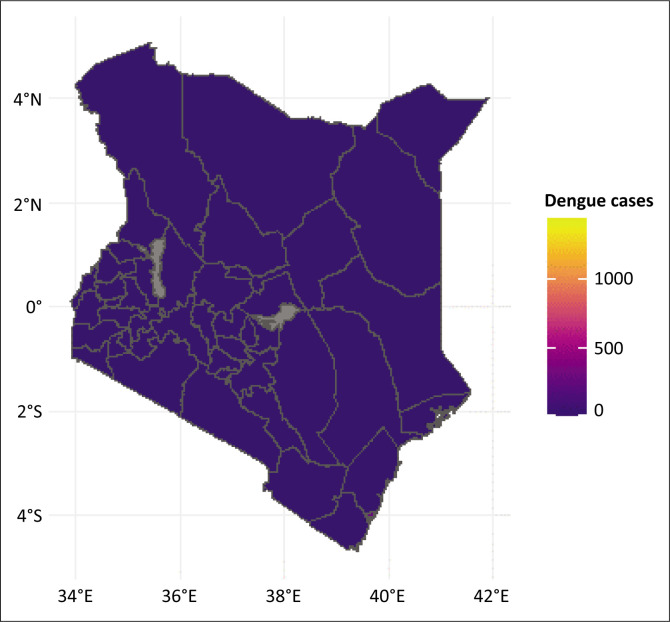
Distribution of dengue cases across Kenyan counties.

Kenya has experienced several significant dengue outbreaks, particularly in coastal regions, since the early 1980s, with notable occurrences in 2011, 2013, and most recently in 2017.^29,30,31,32^ The epidemiology of DF in Kenya is characterised by sporadic outbreaks and variability in disease incidence, including high frequencies of zero counts in some regions and periods (Figure 1). To address these challenges, applying the ZIP model to DF data from Kenya can offer valuable insights into the factors driving disease spread and variability. By analysing data from 2019 to 2021, this study aims to uncover hidden patterns in dengue distribution and identify factors influencing both the occurrence of zero case reports and the variability in non-zero case counts. The findings are expected to enhance our understanding of DF dynamics in Kenya and inform more effective public health strategies and resource allocation.

## Research methods and design

### Study setting

Kenya is a country located in East Africa with its capital city being Nairobi. It spans from approximately 34 °E to 42 °E longitude and from 5 °S to 5 °N latitude (Figure 1). It is bordered by the Indian Ocean to the southeast, providing it with a crucial maritime gateway. The country consists of 47 county governments and one national government, each with distinct roles and responsibilities in health service delivery. The country encompasses a diverse range of geographic features, from coastal plains to highland plateaus and the Great Rift Valley. The total area of Kenya is about 580 367 km^2^, with a population estimated at over 55 million residents in 2023.

**FIGURE 2:**
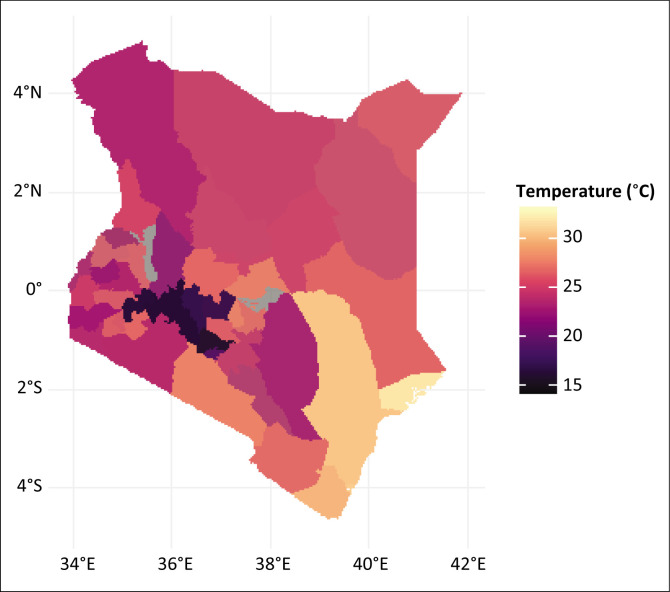
Dengue cases and temperature overlay.

**FIGURE 3:**
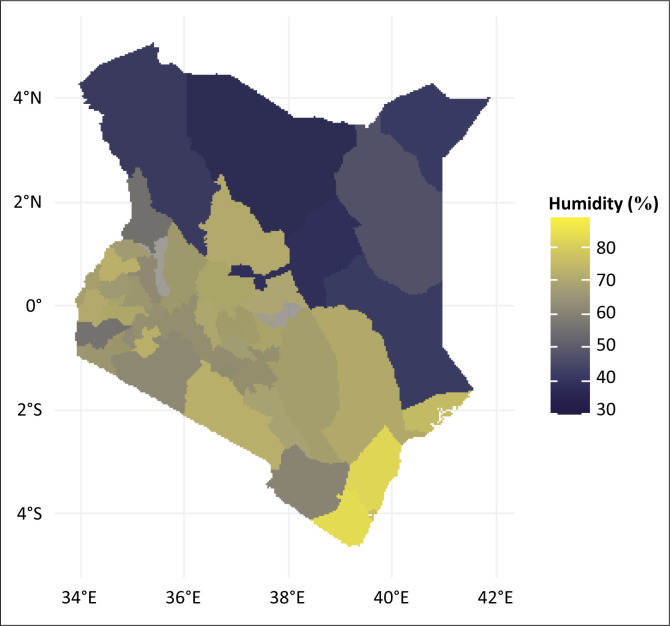
Dengue cases and humidity overlay.

Kenya experiences a range of climates due to its varied topography. The coastal region has a tropical climate with high temperatures (Figure 2) and humidity (Figure 3) throughout the year, while the interior regions experience a more temperate climate. The highlands, including areas like Nairobi and the central region, have a cooler, more temperate climate. The country generally experiences two main rainy seasons: the long rains from March to May and the short rains from October to December. Annual average rainfall ranges from 500 mm in the arid regions to 2000 mm in the highland areas, and average temperatures typically range from 15 °C to 30 °C depending on the region and altitude.

### Data description

Monthly dengue cases in Kenya for the period of 2018–2021 were used in this study. Dengue fever is classified as a notifiable communicable disease in Kenya, with its surveillance and reporting managed according to guidelines established by the Ministry of Health.^33,34^ The diagnosis of DF is based on a combination of epidemiological exposure history, clinical manifestations, and laboratory tests such as white blood cell counts. For cases where the diagnosis is unclear, a specific immunoglobulin G enzyme-linked immunosorbent assay (IgG ELISA) test is conducted to confirm the presence of DENV.^34^ All diagnostic criteria have remained consistent throughout the study period.

To ensure accurate tracking of dengue outbreaks, the Kenyan Ministry of Health has developed a comprehensive reporting system for notifiable diseases. Prior to 2019, data on notifiable communicable diseases were reported manually through paper forms submitted by local health facilities to county health offices and then to the national health authorities.^35,36,37^ In 2019, Kenya transitioned to a digital reporting system through District Health Information Software 2 (DHIS2), which facilitates real-time data submission and management. Data from 2019 to 2021 were extracted from DHIS2.

Monthly weather data, including average minimum temperature (Tmin), average maximum temperature (Tmax), average relative humidity (Hum), and total rainfall (Rain), were obtained from the Kenya Meteorological Department.

### Model formulation

The application of the ZIP model to DF data in Kenya marks a significant advancement in understanding the disease’s epidemiology. Unlike traditional Poisson regression models, which can struggle with excess zeros and over-dispersion in the data, the ZIP model provides a more accurate and comprehensive analysis of dengue cases. This enhanced approach can lead to better predictions of disease outbreaks, more targeted public health interventions, and more efficient allocation of resources. This study aims to leverage the strengths of the ZIP model to gain deeper insights into DF dynamics in Kenya. By addressing the challenges associated with excess zeros and variability in disease data, the research seeks to offer valuable information that can improve public health responses and bolster ongoing efforts to combat DF.

The ZIP regression model is as follows:

Let P_k_ denotes the probability of observing a zero count in the ZIP model. The formula for P_k_ is given by:

$${\text{P}}_{\text{k}}=\frac{exp(y_{i}x_{ki})}{exp(1+y_{i}x_{ki})}$$ [Eqn 1]

Where *y_i_* is the response variable (the count) for the ith observation. *x_ki_* represents the covariates or predictors for the kth term.

The numerator exp(*y_i_x_ki_*) indicates the impact of the covariates on the count data. The denominator exp(1+*y_i_x_ki_*) normalises this to ensure the probability is between 0 and 1. The expected count µ_k_ follows a Poisson distribution with mean µ_k_. It is given by:

$$\mu_{\text{k}}=\exp(\beta_{\text{k}}{\text{x}}_{\text{ki}})$$

Where β_k_ are the parameters associated with the predictors x_ki;_ exp(β_k_x_ki_) models how the predictors influence the mean of the Poisson distribution.

The zero-inflation part of the model uses a logit link function (mixed linear model) to model the probability of extra zeros. The logit function is defined as:

$$\text{Logit }({\text{P}}_{\text{k}})=\log\frac{P_{k}}{1-P_{k}}$$ [Eqn 2]

This function transforms the probability *P_k_* (of observing an extra zero) into a log-odds scale.

The logit function is used to model the binary outcome of whether an observation is an excess zero or not:

$$\begin{array}{l} \text{Logit }({\text{P}}_{\text{k}})=\sum_{k=0}^{j}{\text{y}}_{i}\;{\text{X}}_{\text{ki}}\\ \text{Log}\mu_{\text{k}}=\sum_{k=1}^{j}\beta_{\text{k}}{\text{X}}_{\text{ki}} \end{array}$$ [Eqn 3]

Combining these components, the ZIP model can be expressed as:

$$\begin{array}{l} \text{Logit }({\text{P}}_{\text{k}})=\alpha +\gamma_{\text{ki}}\\ \text{Log(}\mu_{\text{k}})=\beta_{\text{k}}{\text{X}}_{\text{ki}} \end{array}$$ [Eqn 4]

The Logit(P_k_) models the excess zeros using a logit link function. This component determines the probability that an observation is an ‘excess zero’ rather than a count drawn from the Poisson distribution. Log µ_k_ models the actual count data (including zeros) using a Poisson distribution with a mean µ_k_ that depends on the predictors.

### Ethical considerations

Ethical clearance to conduct this study was obtained from the University of Nairobi College of Health Sciences Kenyatta National Hospital Ethics and Research Committee (No. KNH-ERC/A127). The data used in this study were derived from disease surveillance sources, from which all personal identifiers had been permanently removed. As a result, no specific individuals could be identified from the data. Given the anonymised nature of the data, this study did not require further ethics clearance.

## Results

Initially, a descriptive analysis was conducted for all variables to summarise their characteristics (Table 1). The mean number of dengue cases was 456, with a variance of 659, suggesting potential over-dispersion. To formally assess this, we applied Böhning’s over-dispersion test, which compares the sample mean with the sample variance. The O statistic for dengue cases was 456.3, with a *p*-value of 0.004, indicating significant over-dispersion.^38^

**TABLE 1:** Descriptive statistics of key variables and dengue fever cases.

Variable	Mean	Variance	Minimum	Maximum	*O* statistic
Dengue cases	456	659	0	15 000	456.3
Temperature (°C)	25	29	24	30	-
Rainfall (mm)	680	2300	250	2010	-
Humidity (%)	55	76	30	85	-

Note: *p*-value = 0.004.

Furthermore, we employed the Vuong test to determine whether the observed over-dispersion was due to excess zero counts (zero inflation) or true heterogeneity in the data. The results of these tests guided our choice of model, favoring a zero inflated model over a standard Poisson model to account for the excess variability.

In the analysis of DF spread in Kenya using the ZIP modelling approach, an examination of Pearson residuals provides insight into the model’s performance and potential areas for improvement (Table 2).

**TABLE 2:** Pearson residuals.

Statistic	Value
Minimum	−0.1916
1st Quartile	−0.1913
Median	−0.1909
3rd Quartile	−0.1903
Maximum	24.6637

The median Pearson residual is –0.1909; it is close to zero, suggesting that for the majority of observations, the ZIP model’s predictions are relatively accurate. This indicates that the model effectively captures the general trend in DF incidence across the dataset, providing a reasonable fit for most locations and time points. The observed maximum residual is 24.6637 which is notably high, indicating significant discrepancies between the observed dengue cases and those predicted by the model in certain instances. This extreme positive residual points to cases where the model under-predicts dengue cases substantially. Such outliers highlight specific scenarios where the ZIP model’s assumptions or covariates might not fully capture the variability in the data.

The results from the Poisson regression model indicate that temperature and humidity have significant positive effects on dengue cases, while rainfall has a significant negative effect. These findings are presented in Table 3.

**TABLE 3:** Count model coefficients (Poisson with log link).

Variable	Intercept	Std. error	*Z*-values	Pr(>|*z*|)
(Intercept)	0.1109	0.1515	0.7320	0.464
Temperature	0.0558	0.0053	10.4970	< 2e-16
Rainfall	−0.0045	0.0003	−16.5230	< 2e-16
Humidity	0.0578	0.0024	24.1570	< 2e-16

Std., standard.

The intercept of the Poisson regression model is estimated at 0.1109, with a standard error of 0.1515. The z-value of 0.732 and the associated *p*-value of 0.464 indicate that the intercept is not statistically significant. This implies that the baseline log count of dengue cases, when all predictor variables are at their reference levels, is not significantly different from zero. However, because temperature, rainfall, and humidity cannot realistically be zero, the intercept’s interpretability in isolation is limited.

The coefficient for *temperature* in Table 3 is 0.0558 (standard error [s.e.] = 0.0053, *z* = 10.497, *p* < 2e-16), indicating a highly statistically significant positive association with dengue cases. Specifically, a 1 °C increase in temperature is associated with an expected increase of approximately ‘0.056 dengue cases per month’. Scaling this effect, a 10 °C rise in temperature would lead to an increase of approximately ‘0.56 cases per month’, highlighting the critical role of rising temperatures in exacerbating dengue transmission risk.

For ‘rainfall’, the coefficient is –0.0045 (s.e. = 0.0003, *z* = –16.523, *p* < 2e-16), also highly statistically significant. This negative relationship suggests that for every 1 mm increase in rainfall, there is an expected reduction of approximately ‘0.0045 dengue cases per month’. Over a larger scale, an increase in rainfall by 100 mm would reduce dengue cases by about ‘0.45 per month’. While counter-intuitive, this inverse relationship could be explained by the disruptive effects of heavy rains on mosquito breeding sites, which may decrease mosquito populations and thereby lower dengue transmission.

The coefficient for ‘humidity’ is 0.0578 (s.e. = 0.0024, *z* = 24.157, *p* < 2e-16), showing a strongly significant positive relationship. This implies that a 1% increase in humidity corresponds to an expected rise of approximately ‘0.058 dengue cases per month’. Over a larger scale, a 10% increase in humidity would result in an additional ‘0.58 cases per month’. This finding underscores the role of high humidity in enhancing mosquito survival and increasing the potential for DENV transmission.

The findings indicate that temperature and humidity have a positive effect on dengue cases, while rainfall has a negative impact. These relationships emphasise the importance of climatic conditions in influencing the spatial and temporal dynamics of DF.

The significant zero-inflation component supports the use of the ZIP model over a standard Poisson model, as it accounts for the excess zeros that are systematically present in the data. This feature of the ZIP model is crucial for accurately representing and analysing the distribution of dengue cases, as it provides a more nuanced understanding of both the count of cases and the occurrence of zero counts in the dataset.

The intercept (Table 4) for the zero-inflation component of the model is estimated at 3.3032, with a standard error of 0.2277. The *z*-value of 14.51 and the *p*-value less than 2e-16 indicate that this coefficient is highly statistically significant (Table 4). The positive and significant intercept in the zero-inflation model suggests a high likelihood of observing excess zeros in the dataset. In other words, many observations in the data have zero dengue cases more frequently than would be expected by a standard Poisson distribution alone. This indicates that the zero-inflation component is effectively capturing a structural characteristic of the data where certain locations or periods consistently report no dengue cases. The high intercept value implies that these excess zeros are not just random fluctuations but reflect a systematic aspect of the data. This could be due to several factors such as non-endemic areas where DF does not occur, effective control measures that prevent outbreaks, or other factors leading to a consistent absence of cases (Table 5).

**TABLE 4:** Zero-inflation model coefficients (Binomial with logit link).

Variable	Intercept	Std. error	*Z*-score	Pr(>|*z*|)
(Intercept)	3.3032	0.2277	14.51	< 2e-16

Std., standard.

**TABLE 5:** Model fit and comparison.

Model type	AIC	Log-likelihood
ZIP model	5230.959	0.8070
Poisson model	27061.367	N/A

ZIP, Zero-Inflated Poisson; AIC, akaike information criterion; N/A, not applicable

The model fit statistics for the ZIP model and the standard Poisson model are summarised in Table 5. The ZIP model has an akaike information criterion (AIC) value of 5230.959 and a log-likelihood of 0.8070. In contrast, the Poisson model exhibits a substantially higher AIC value of 27061.367, with the log-likelihood value not applicable (N/A) due to its higher complexity in this context.

The AIC is a measure used for model comparison, where a lower AIC value indicates a better fit when accounting for model complexity. The substantial difference between the AIC values for the ZIP and Poisson models suggests that the ZIP model provides a significantly better fit for the data. This is particularly relevant given the presence of excess zeros in the dataset, which the ZIP model is specifically designed to handle.

The log-likelihood value for the ZIP model, which is positive at 0.8070, indicates that the model fits the data well, especially in capturing the distribution of dengue cases and the excess zeros. Although the Poisson model’s log-likelihood was not available, its much higher AIC value further supports that the ZIP model’s fit is superior.

The ZIP model not only demonstrates a significantly better fit to the data as evidenced by the lower AIC value but also effectively addresses the zero-inflation present in the dataset. This makes it the preferred model for analysing DF cases in this study, as it provides a more accurate and reliable representation of the underlying data structure.

## Discussion

This study used the ZIP model to investigate the impact of climatic factors on DF incidence in Kenya from 2019 to 2021. The ZIP model proved effective in handling the over-dispersion and excess zeros present in the data, offering a more nuanced understanding of dengue dynamics compared to traditional Poisson models.

Our findings reveal that temperature and humidity have a positive relationship with dengue cases, while rainfall shows a negative effect. Specifically, the coefficient for temperature suggests that as temperatures rise, the incidence of DF increases. This result aligns with other research that warmer temperatures accelerate mosquito development and increase their activity, thereby enhancing dengue transmission.^14,16,17,18,19,22^ Similarly, the positive association with humidity supports the understanding that higher humidity levels create favourable conditions for mosquito survival and DENV transmission.^16,22,23^

On the other hand, the negative relationship between rainfall and dengue cases is less spontaneous. Increased rainfall usually creates more breeding sites for mosquitoes, which would typically be expected to increase dengue incidence. However, this study’s findings might be explained by the possibility that heavy rains disrupt mosquito breeding habitats or that effective vector control measures were in place during the study period.^15,19,22,39,40,41^ Such factors could have mitigated the impact of rainfall on dengue transmission.

The ZIP model’s performance, indicated by its lower AIC and higher log-likelihood compared to the standard Poisson model, underscores its suitability for this analysis. The significant excess zeros in the dataset make the ZIP model a better fit, as it accounts for the zero-inflation that traditional Poisson models fail to address. This improved model fit confirms the ZIP model’s ability to accurately represent the distribution of dengue cases and better inform public health strategies.

The findings underscore the importance of climate-informed strategies in managing dengue outbreaks. The positive associations between temperature, humidity, and dengue incidence suggest that public health interventions could be timed and scaled based on climate forecasts, particularly in warmer, more humid months when transmission risk is highest. By understanding these environmental triggers, health authorities can prioritise resources for mosquito control, public awareness campaigns, and healthcare preparedness in areas expected to experience high temperatures and humidity.

The insights from the ZIP model allow for more nuanced predictions by accounting for both the occurrence and count of dengue cases. This can help health authorities develop proactive, data-driven responses that not only predict when outbreaks are likely to happen but also address the intensity of potential outbreaks. By integrating climate variables into surveillance systems, regions can adapt quickly, deploying preventive measures when conditions are most conducive to dengue spread, which ultimately enhances the efficiency of public health interventions.

### Limitations

The reliance on secondary data from the Kenyan Ministry of Health introduces potential issues related to data accuracy and completeness. Variations in data quality affect the reliability of the results. Additionally, while the ZIP model addresses zero-inflation, its assumptions may not fully capture all aspects of the data. Further exploration of alternative models or additional covariates might be necessary to refine the analysis.

The geographic and temporal scope of the study focusing on Kenya from 2019 to 2021 did not encompass long-term trends and regional variations. Expanding the analysis to cover a broader time frame and additional regions could provide a more comprehensive view of DF dynamics. Moreover, the use of average monthly weather data may overlook micro-climatic variations or short-term fluctuations that could influence dengue transmission. Incorporating more granular climatic data could enhance the model’s accuracy.

## Conclusion

The ZIP model has provided valuable insights into the relationship between climatic variables and DF incidence in Kenya. Th ZIP model addresses the excess zeros and over-dispersion in the data. This has led to enhanced understanding of how temperature and humidity influence DF, while offering a more nuanced perspective on the impact of rainfall.

Future research should aim to address the limitations identified in this study. Improved data accuracy, broader geographic and temporal coverage, and the integration of more detailed climatic data could further refine the understanding of DF dynamics. Such efforts will contribute to more targeted interventions and enhanced strategies for combating DF in Kenya and other affected regions.
